# Methodological Challenges in Randomized Controlled Trials on Smartphone-Based Treatment in Psychiatry: Systematic Review

**DOI:** 10.2196/15362

**Published:** 2019-10-27

**Authors:** Morten Lindbjerg Tønning, Lars Vedel Kessing, Jakob Eivind Bardram, Maria Faurholt-Jepsen

**Affiliations:** 1 Copenhagen Affective Disorder Research Center Psychiatric Center Copenhagen, Rigshospitalet University Hospital of Copenhagen Copenhagen Denmark; 2 Department of Health Technology Technical University of Denmark Lyngby Denmark

**Keywords:** psychiatry, methodology, smartphone, mHealth, mobile Health, digital health, digital psychiatry, systematic review

## Abstract

**Background:**

Smartphone-based technology is developing at high speed, and many apps offer potential new ways of monitoring and treating a range of psychiatric disorders and symptoms. However, the effects of most available apps have not been scientifically investigated. Within medicine, randomized controlled trials (RCTs) are the standard method for providing the evidence of effects. However, their rigidity and long time frame may contrast with the field of information technology research. Therefore, a systematic review of methodological challenges in designing and conducting RCTs within mobile health is needed.

**Objective:**

This systematic review aimed to (1) identify and describe RCTs investigating the effect of smartphone-based treatment in adult patients with a psychiatric diagnosis, (2) discuss methodological challenges in designing and conducting individual trials, and (3) suggest recommendations for future trials.

**Methods:**

A systematic search in English was conducted in PubMed, PsycINFO, and EMBASE up to August 12, 2019. The search terms were (1) psychiatric disorders in broad term and for specific disorders AND (2) smartphone or app AND (3) RCT. The Consolidated Standards of Reporting Trials electronic health guidelines were used as a template for data extraction. The focus was on trial design, method, and reporting. Only trials having sufficient information on diagnosis and acceptable diagnostic procedures, having a smartphone as a central part of treatment, and using an RCT design were included.

**Results:**

A total of 27 trials comprising 3312 patients within a range of psychiatric diagnoses were included. Among them, 2 trials were concerning drug or alcohol abuse, 3 psychosis, 10 affective disorders, 9 anxiety and posttraumatic stress disorder, 1 eating disorder, and 1 attention-deficit/hyperactivity disorder. In addition, 1 trial used a cross-diagnostic design, 7 trials included patients with a clinical diagnosis that was subsequently assessed and validated by the researchers, and 11 trials had a sample size above 100. Generally, large between-trial heterogeneity and multiple approaches to patient recruitment, diagnostic procedures, trial design, comparator, outcome measures, and analyses were identified. Only 5 trials published a trial protocol. Furthermore, 1 trial provided information regarding technological updates, and only 18 trials reported on the conflicts of interest. No trial addressed the ethical aspects of using smartphones in treatment.

**Conclusions:**

This first systematic review of the methodological challenges in designing and conducting RCTs investigating smartphone-based treatment in psychiatric patients suggests an increasing number of trials but with a lower quality compared with classic medical RCTs. Heterogeneity and methodological issues in individual trials limit the evidence. Methodological recommendations are presented.

## Introduction

### Background

Psychiatric disorders represent a major burden of disease worldwide with a significant impact on the quality of life, socioeconomic factors, and life expectancy [1]. In 2010, the worldwide expenses because of mental illness were estimated to be between US $2.5 trillion and US $8.5 trillion [2]. Across European countries, 27% of the adult population suffers from at least one psychiatric disorder [3]. At the same time, there is a gap between the need for treatment and the number of patients receiving treatment. The number of patients who do not receive treatment for their disorder is 35% to 50% in high-income countries and 76% to 85% and in low- and middle-income countries [4].

In 2011, the World Health Organization stated that “the use of mobile and wireless technologies to support the achievement of health objectives (mHealth) has the potential to transform the face of health service delivery across the globe” [5]. The number of smartphone users exceeded 2.5 billion people in 2018 [6], and in high-income countries, 80% of the population own and use a smartphone [7].

Smartphones are a promising tool in the field of psychiatry. They are widely used and always at hand, allowing for delivery of treatment to patients in real-time and naturalistic settings, and can augment already available treatments. At the same time, smartphones contain several sensors and technologies enabling patients, researchers, and clinicians to access information about physical and social activities [8].

Mobile health (mHealth) and especially smartphone-based technology and solutions are developing at an enormous speed, driven mainly by software and computer scientists and private companies. Thus, most available apps have not been scientifically investigated, and the validity, treatment effect, and safety have been sparingly investigated [9,10]. Nevertheless, hundreds of apps claiming to help or monitor psychiatric disorders are already available in app stores [11].

### Evidence, Randomized Controlled Trials, and Interdisciplinary Research

Randomized controlled trials (RCTs) represent the methodological golden standard of excellence in medical research for the investigation of possible positive and negative effects of treatment interventions [12]. The importance of RCTs in medical science is mainly because of their ability to eliminate confounding of known and unknown nature. If properly designed and conducted, RCTs are especially useful for examination of small or moderate positive and negative effects [12].

The design and conduct of RCTs within smartphone-based treatment interventions and other electronic treatments should follow the RCT standards within medicine, while also taking into account particular challenges with electronic effect research. In RCTs, in general, there is a significant time gap from design and trial initiation to the publication of results. A recent Australian study of publicly funded clinical trials showed a median time of 7.1 years (95% CI 6.3-7.6 years) from funding to the main article on trial results being published [13]. This time gap is particularly problematic when testing smartphone-based treatment interventions as the technological development taking place within the timespan of an RCT is enormous. The technology tested is at risk of being outdated when results are published and before being taken into clinical usage [14]. Furthermore, the locked nature of treatment interventions tested in RCTs contrasts with the constantly evolving and iterative nature of app solutions [15].

### Previous Reviews

Other groups have previously suggested changes to the development process, to speed up the process from idea to publication [14,16,17]. A previous review from 2013 [18] identified effect studies of smartphone-based treatments within psychiatry (including stress). This review was not limited to include RCTs, and thus, it also included pre- to posttest studies. Only 8 studies were identified despite an extensive search and broad inclusion criteria.

Symptom-specific reviews within branches of psychiatry have been done as well. As an example, Firth et al [19] found studies regarding depressive *symptoms* and conducted a meta-analysis on the possible effects of smartphone-based treatments on these symptoms. A total of 18 RCTs were identified; however, often, a psychiatric diagnosis was not present as the focus was on depressive symptoms. This study focused on RCTs only. The focus was on research methods and design in relation to smartphone-based treatment interventions within psychiatry. This study was limited to RCTs providing information on diagnostic measures to ensure that participants suffers from a psychiatric diagnosis, excluding trials focusing on symptoms in healthy populations.

### Aims

This systematic review aimed to identify and describe all available RCTs using smartphones as (part of) a treatment intervention conducted in the field of psychiatry using sufficient diagnostic measures. Furthermore, it aimed to describe the methodology of these individual trials and discuss methodological challenges related to designing and conducting RCTs within smartphone-based treatment in psychiatric disorders using the Consolidated Standards of Reporting Trials (CONSORT) electronic health (eHealth) checklist [20] as a reference and to provide recommendations for future trials within the area.

## Methods

Methods of the review and eligibility criteria were established in advance by 3 of the authors (MLT, MFJ, and LVK). Minor modifications such as adding further information to be extracted were made to the review protocol during the review process.

### Trial Selection

Original trials reporting on smartphone-based treatment interventions investigated in RCTs, including adult patients with psychiatric disorders, were eligible for review. In addition, peer-reviewed articles, posters, and conference abstracts were eligible for review. If multiple articles reported on overlapping populations, the article presenting the largest population was included. No restrictions regarding sample size were applied.

The exclusion criteria were as follows: (1) children younger than 18 years; (2) psychiatric symptoms as part of somatic disorders (ie, preoperation anxiety or depressive symptoms in terminal cancer patients); (3) trials concerning stress, cigarette smoking, low intelligence quotient (IQ), and isolated sleep problems without psychiatric disorders; (4) trials with individuals who self-identified as having a psychiatric diagnosis but without diagnostic reassurance; (5) trials reporting on symptoms without diagnoses (ie, depressive symptoms or alcohol usage among college students); (6) trials using internet therapy without an active smartphone-based component (ie, if the Web page was accessible from a smartphone browser); (7) trials using only cell phones in traditional ways with text messages and phone calls (not using smartphone-based features); (8) trials using smartphones as a screen for virtual-reality setups as the primary treatment component; and (9) trials not available in English.

### Information Source, Trial Selection, and Data Extraction

We conducted a systematic search covering PubMed, PsycINFO, and EMBASE on April 23, 2018, and it was last updated on August 12, 2019. Only articles from 2008 onwards were included (the time of the release of the first smartphone). References from articles and other reviews were also examined, but they did not result in any additional trials to include. The trial selection was conducted by 2 researchers (MFJ and MLT), and articles with doubt about the relevance were discussed between the 2 of them. Full-text articles for possible relevant trials were obtained if the abstract and title did not supply sufficient information. The search strategy included (1) psychiatric disorder as a broad term and for specific diseases AND (2) smartphone or app AND (3) RCT. A wide variety of text words were used to include trials published within the last 6 months that had not yet been indexed with Medical Subject Headings terms. Search strategy in PubMed was as follows:

(((Smartphone[MeSH terms] OR mobile application[MeSH Terms] OR smartphone OR mobile application OR smart phone OR mobile phone OR app OR apps OR cell phone OR Iphone* OR IOS OR Android phone OR smartphones OR mobile applications OR smart phones OR mobile phones OR cell phones)) AND (((((((((mental disorder[MeSH Terms]) OR (mental disorder OR mental disorders OR mental disease OR mental diseases OR mental diagnose OR psychiatric disease OR psychiatric diseases OR psychiatric disorders OR psychiatric disorder OR psychiatric diagnose)) OR ((drug OR substance OR prescription drug OR alcohol OR narcotic OR heroin OR amphetamine OR cocaine OR marijuana OR opioid OR morphine OR phencyclidine) AND (abuse OR dependence OR addiction))) OR (feeding disorder OR feeding disorders OR eating disorders OR eating disorder OR anorexia OR bulimia OR binge eating)) OR (autism OR autistic OR Asperger disease OR Aspergers disease) OR Asperger disorder OR Aspergers disorder OR ADHD OR attention deficit disorder OR ADD OR attention deficit hyperactivity disorder)) OR (personality disorder OR personality disorders OR obsessive-compulsive personality OR compulsive personality OR obsessive personality OR psychopath OR sociopathic OR antisocial OR passive-dependent personality OR dyssocial OR schizoid OR schizotypal)) OR (schizophrenia OR psychoses OR psychosis OR psychotic OR paranoid OR schizoaffective OR schizophreniform OR delusional)) OR (major depressive disorder OR unipolar depression OR unipolar disorder OR depressive syndrome OR endogenous depression OR neurotic depression OR melancholia OR cyclothymic OR dysthymic OR mood disorder OR mood disorders OR affective disorder OR affective disorders OR bipolar OR manic-depressive OR mania OR manic) OR (anxiety OR anxieties OR panic disorder OR agoraphobia OR obsessive disorder OR compulsive disorder OR obsessive-compulsive disorder OR phobic disorder OR phobic disorders OR PTSD OR posttraumatic stress disorder OR post-traumatic stress disorder OR post traumatic stress disorder))) AND ((randomized controlled trial[MeSH Terms]) OR (randomized controlled trial OR randomised controlled trial OR randomised OR randomized OR RCT OR randomized clinical trial OR randomiced clinical trial OR randomized clinical trial OR randomized controlled clinical trial OR randomised controlled clinical trial))

Data were extracted by using the CONSORT eHealth checklist as a template for data extraction [20]. The following data were extracted:

- Author, year, country, trial design, trial registration, protocol availability, patients’ age and gender, sample size and use of power calculations, length of treatment intervention, and follow-up period.
- Recruitment and diagnostics procedures of patients, recruitment length, outcome measures, well-defined hierarchy of outcome measures, and collection of outcome data.
- Description of treatment intervention and comparator, use of blended treatment and standard treatments, affiliation with industry and technology descriptions.
- Title according to CONSORT recommendations; use of prompts, platform choice, and possible lent-out of smartphones; economic compensation; use of placebo smartphones; methodological information regarding randomization and blinding procedures; information about the statistical approach to technical updates and whether updates and technical crashes or down periods were reported; possible harms; adherence to the smartphone system; and baseline data on patients’ technological skills and funding information.

A data extraction template is provided in the supplementary material (Multimedia Appendix 1). The extracted data are presented in 4 tables. Data were initially retrieved by one author (MLT) and subsequently and independently by another author (MFJ). Any disagreements were solved between MLT and MFJ.

Three tables describe the trials individually, with various focus. The fourth uses the CONSORT eHealth checklist [20] as a template to summarize the relevant findings according to these in a systematic way and hereby partly summarizes the relevant findings from previous tables, but it also includes other new relevant information. The tables are presented in relevant sections in the result-section.

## Results

### Trial Selection

**Figure 1:**
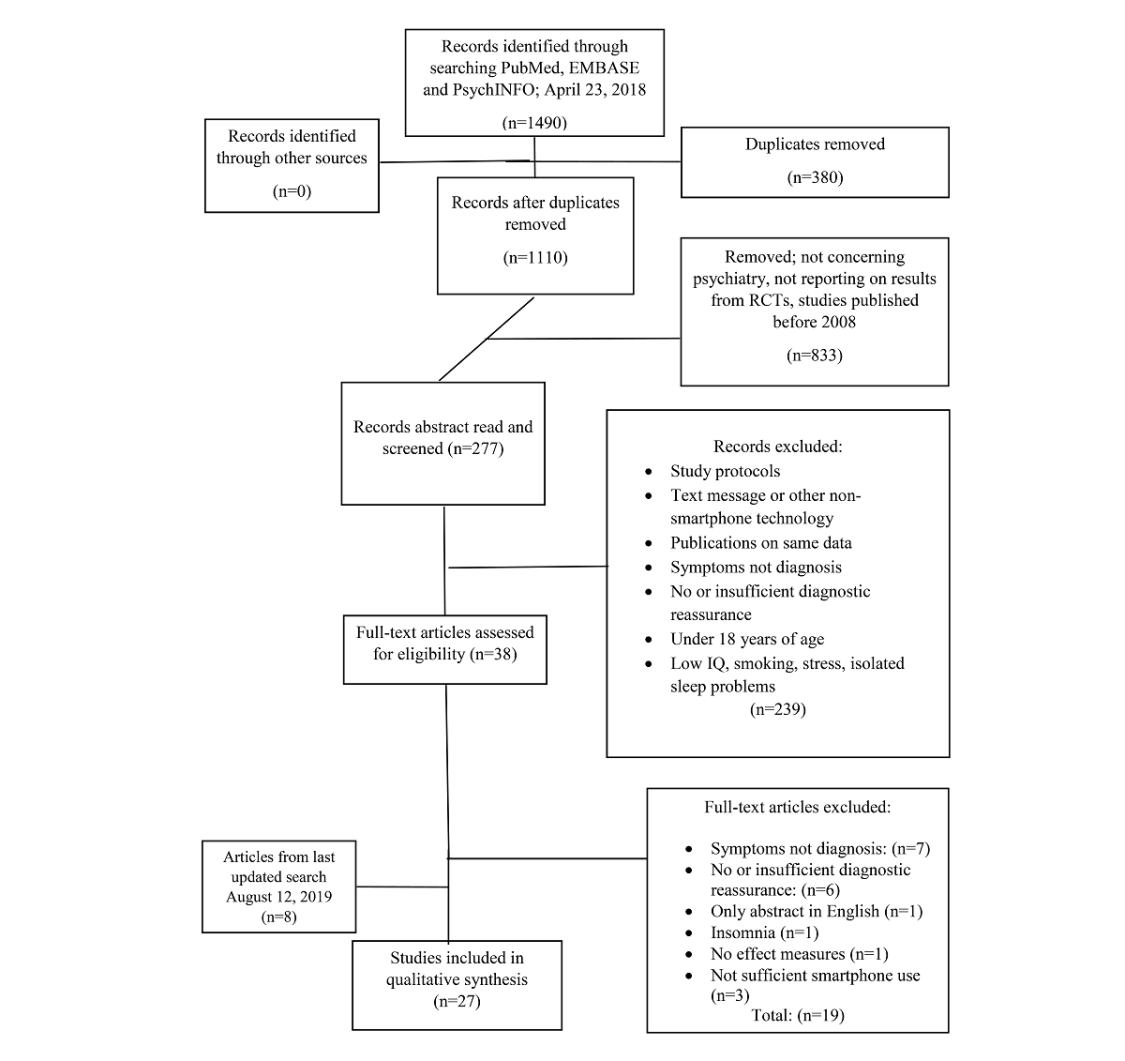
Preferred Reporting Items for Systematic Reviews and Meta-Analyses flow diagram displaying information on article flow from initial search to final inclusion. IQ: intelligence quotient; RCT: randomized controlled trial.

Figure 1 presents a flow diagram represented according to the Preferred Reporting Items for Systematic Reviews and Meta-Analyses guidelines [21], showing the results of the literature search and selection of trials. The initial literature search in PubMed, EMBASE, and PsycINFO on April 23, 2018, resulted in a total of 1490 articles. Of these, 380 duplicates were removed, resulting in 1110 articles. Furthermore, 833 articles were excluded based on the title and year of publication with the main reasons for exclusion being as follows: not concerning psychiatric disorders (eg, HIV, Alzheimer disease, and obesity), not reporting on results from RCTs, and published before 2008. This led to a total of 277 remaining articles, from which abstracts were examined.

Out of these 277 articles, 239 were excluded based on abstracts, with majority of reasons being as follows: trial protocols, text message or other nonsmartphone technology, publications on same data (the publication with the most data was included), measuring on symptoms—not diagnosis, no or insufficient diagnostic reassurance, participants younger than 18 years, topics concerning low IQ, smoking, stress, or isolated sleep problems.

This led to 38 articles that were printed for full-text reading. Among them, 19 articles were subsequently excluded because of the following: reporting symptoms, not disorders (n=7); no or insufficient diagnostic reassurance (n=6); only abstract in English (n=1); insomnia (n=1); no outcome effect measures (n=1); and not using smartphone as part of treatment (ie, only using smartphones to reach the patient or allowing patients to answer emails and questionnaires on smartphone; n=3). References from articles and other reviews were also examined, but they did not result in any additional trials to include, giving a total of 19 eligible articles from the initial search.

The search was updated on August 12, 2019, resulting in 8 new articles. Thus, a total of 27 unique trials [22-48] were identified and included in this review, including 5 with diagnostic estimates based on questionnaires using relevant cutoff scores [44-48]. Included trials are described individually in the tables.

### Trial Characteristics

Across all included trials, a total of 3,312 patients were included. The included trials represented a range of psychiatric disorders. Numbers in parentheses represent relevant chapter and coding according to International Statistical Classification of Diseases and Related Health Problems-10 (ICD-10): 2 on drug or alcohol misuse (F10-F19) [24,37], 3 on psychosis (F20-29) [35,38,41], 10 on affective disorders (F30-39) [22,25-28,32,36,40,42,45] (comprising 3 on bipolar disorder and 7 on unipolar depressive disorder), 9 on anxiety and posttraumatic stress disorder posttraumatic stress disorder (F40-F48) [23,30,34,39,43,44,46-48], 1 on eating disorders (F50-F59) [31], and 1 on attention-deficit disorder (F90-F98) [29]. One trial included patients with severe mental disorders across ICD-10 diagnoses [33].

Most trials had an overrepresentation of females, except trials concerning veterans, schizophrenia, or substance or alcohol abuse. Patients’ ages ranged from 18 to 73 years. In the majority of the trials, the average age was 40 years.

### Trial Design and Reporting

The number of RCTs testing smartphone solutions in patients with a psychiatric disorder increased with time, especially from 2018 onward. Some trials [24,27,29-33,35,40] were leaning toward traditional RCT designs conducted in clinical settings and using the CONSORT checklist for reporting and designing trials [20]. Some other trials did not provide clear information regarding these issues, leaving the reader with lacking information on the design and conduct of the trial.

Overall, 11 trials had a sample size above 100 patients [24,30,32-34,39-41,44,45,48]. One trial [40] had been repeated using the same intervention, trial design, and outcome measures as a previous trial [27].

A total of 14 articles included information about trial registration, such as the clinicaltrials.gov [22,24-27,29-33,35,39-41]. For the remaining trials (including the study by Krzystanek et al [41], where we could not find any information on the registration information provided) it was not possible to see whether the primary outcome measure was predefined in the primary hypothesis leading to the trial design. In addition, 5 trials referred to a published trial protocol in the article [27,30,32,35,40]. One additional article attached the study protocol in the supplementary section when published [28].

Information about the individual trial designs, registration, sample characteristics, and length of intervention and follow-up is presented in Table 1.

**Table 1:** Randomized controlled trials involving smartphones in the field of psychiatry identified in systematic search updated in August 2019. Description of basic information and trial design. The bottom 5 trials in *italics* are trials with diagnoses solely based on questionnaires.

Author, year of publication	Country	Trial design	Protocol^a^	Female/total	Analyzed (power calculation)	Age (years), mean (SD)	Intervention length (weeks)	Posttreatment follow-up (weeks)
Watts et al, 2013 [22]	Australia	Pilot RCT^b^	N/A^c^	28/35	25 (N/A)	41 (12.4)	8	12
Dagöö et al, 2014 [23]	Sweden	RCT	N/A	39/57	52 (N/A)	52	9	12
Gustafson et al, 2014 [24]	United States	RCT	N/A^c^	137/349	349 (350)	38 (10)	32	16
Ly et al, 2014 [25]	Sweden	RCT	N/A^c^	57/81	81 (N/A)	36.0 (10.8)	8	16
Depp et al, 2015 [26]	United States	RCT	N/A^c^	48/82	82 (N/A)	47.5	10	14
Faurholt-Jepsen et al, 2015 [27]	Denmark	RCT	Published^c^	45/67	78 (56)	18-60^d^	24	No
Ly et al, 2015 [28]	Sweden	NI^e^ RCT	Attached	65/95	93 (93)	18-73^d^	9	24
Moëll et al, 2015 [29]	Sweden	RCT	N/A^c^	39/57	57 (N/A)	36.8 (10.9)	6	No
Ivanova et al, 2016 [30]	Sweden	RCT^f^	Published^c^	98/152	152 (150)	35.3	10	52
Hildebrandt et al, 2017 [31]	United States	RCT	N/A^c^	55/66	66 (80)	32.1	12	24
Mantani et al, 2017 [32]	Japan	RCT	Published^c^	87/164	164 (164)	25-59^d^	8	17
Ben-Zeev et al, 2018 [33]	United States	RCT	N/A^c^	67/163	163 (160)	49 (10)	12	12
Boettcher et al, 2018 [34]	Sweden/Germany	RCT^f^	N/A	161/209	209 (N/A)	35.4 (12.4)	12	52
Bucci et al, 2018 [35]	England	Pilot RCT	Published^c^	18/36	36 (N/A)	N/A	12	10
Hur et al, 2018 [36]	Republic of Korea	Pilot RCT	N/A	26/48	34 (N/A)	≈24	3	No
Liang et al, 2018 [37]	China	Pilot RCT	N/A	21/74	75 (N/A)	41.6 (8.0)	4	No
Schlosser et al, 2018 [38]	United States	RCT	N/A	15/43	43 (N/A)	24	12	12
Stolz et al, 2018 [39]	Switzerland	RCT^f^	N/A^c^	94/150	150 (141)	35	12	12
Faurholt-Jepsen et al, 2019 [40]	Denmark	RCT	Published^c^	76/129	129 (117)	43 (12.4)	36	No
Krzystanek et al, 2019 [41]	Poland	RCT	N/A^c^	116/290	Varying	32.1 (6.2)	52	No
Stiles-Shields et al, 2019 [42]	United States	Pilot RCT	N/A	N/A/30	27 (N/A)	N/A	6	4
Teng et al, 2019 [43]	Taiwan	RCT^f^	N/A	61/82	82 (N/A)	≈21.5	4	4
*Enock et al, 2014 [44]*	United States	RCT	N/A	205/429	326^g^ (N/A)	18-68^d^	4	8
*Roepke et al, 2015 [45]*	United States	RCT^f^	N/A	197/283	283 (207)	40.2 (12)	4	2
*Miner et al, 2016 [46]*	United States	Pilot RCT	N/A	40/49	49 (N/A)	45.7 (13.9)	4	4
*Possemato et al, 2015 [47]*	United States	Pilot RCT	N/A	1/20	20 (N/A)	42	8	No
*Kuhn et al, 2017 [48]*	United States	RCT	N/A	83/120	120 (120)	39	12	12

^a^Mentioned in the article.

^b^RCT: randomized controlled trial.

^c^Trial is registered.

^d^Age interval (mean not given in the article).

^e^NI: noninferiority.

^f^The trial had 3 arms.

^g^Included in the analysis if score is greater than cutoff.

### Settings and Diagnostic Procedures

A total of 8 trials were conducted in traditional clinical settings, with patients referred by clinical staff or contacted in their treatment clinic [24,27,32,33,35,37,40,41]. Others used remote designs where patients who self-identified as having a psychiatric disorder applied for participation [22,23,25,26,28,30,34,38,39,42-44,46,48]. None of the trials compared participants with nonparticipants. All but 2 trials [43,44] presented a flowchart of eligible subjects with varying details on reasons not to participate. Trials with open recruitment (eg, internet forums, Web pages, Facebook, and advertising) [22,23,25,26,28-30,34,36,38,39,42-46,48] only had information on people who signed up. Completion rates were reported very differently and varied from 163 patients out of 164 (99.4% [163/164]) completing the primary outcome [32] to 74 out of 283 patients (26.1% [74/283]) [45]. In addition, 12 trials compensated for participation in trial assessments with money or gift cards [26,33-38,42,43,46-48].

Validation and certainty of diagnosis varied substantially, and often, a pragmatic setup was used, leaving the validity of the obtained diagnosis with uncertainty.

A total of 5 trials based their diagnosis solely on clinical-based information [24,33,35,37,41]. Furthermore, 15 trials used research-based diagnoses without clinical information, using either questionnaires [44-46,48], phone calls [22,23,25,28,34,42], video interviews [38], or personal interviews [31,36,39,43] to validate the self-reported diagnoses. In addition, 7 trials included patients with a clinical diagnosis that was subsequently assessed and validated by the researchers using questionnaires [47], phone calls [29,30], or personal interviews [26,27,32,40].

### Hypotheses and Use of Predefined Hierarchical Outcome Measures

Overall, 18 trials included clearly described hypotheses [22,24-28,30-32,34,36,38-40,44,45,47,48], and 8 trials [22,37,41-44,46,47] did not distinguish between primary and secondary outcome measures in the article and had no hierarchy of outcomes.

### Outcome Measures

A total of 8 trials included objective or observer-based primary outcome measures: One trial tested the levels of drug use in the urine as a specific primary outcome measure [37]. Another trial used objective measurements of feasibility, use, and attrition as the primary outcome [35]. One trial tested a specific task of motivated behavior [38]. In addition, 4 trials used scores on clinical ratings as the primary outcome measure assessing the level of depression and mania [26,27,40], levels of bulimic episodes [31], or psychotic symptoms [41]. The remaining trials used patient-reported outcome measures.

Within trials of similar diagnoses, authors used different scales and measures for the primary outcome measure. For instance, in measuring depression scores, the following outcome measures were used as the primary outcome: Patient Health Questionnaire, Beck Depression Inventory, Hamilton Rating Scale for Depression 17 item, Montgomery–Åsberg Depression Rating Scale, and Center for Epidemiologic Studies Depression Scale [22,25-28,32,33,36,40,42,45].

Furthermore, 2 trials, with apps for training in attention bias modification, claimed to be double blinded, with no further explanation on how blinding was ensured [43,44]. One trial blinded app allocation for patients when testing 2 different apps [45]. The remaining trials did not blind patients for the intervention (active vs control). In 12 trials, the authors explicitly stated that they had used blinded assessments for outcome measures [25-29,31-33,35,38,40,44]. Within these 12 trials, 5 trials used the patient-reported outcome as the primary outcome measure in nonblinded patients [25,28,29,32,33]. Although blinded assessors collected these data, answers to the questionnaires were self-reported. One trial tested for the success of blinding [32].

Information about recruitment, diagnosis, outcome measures, and how these were obtained can be found in Table 2.

**Table 2:** Randomized controlled trials involving smartphones in the field of psychiatry identified in systematic search updated in August 2019. Description of participant characteristics and outcome data. The bottom 5 trials in *italics* indicate trials with diagnoses solely based on questionnaires.

Author, year of publication	Diagnosis	How was the diagnosis obtained?	Recruitment: *open/closed (recruitment length in months);* information	Primary outcome^a^	Questionnaire data collection
Watts et al, 2013 [22]	Major depressive disorder (R^b^)	MINI^c^ phone interview + PHQ-9^d^	*Open (3 months);* advertising + application on a Web page	*Questionnaire***(**PHQ-9, BDI-II^e^, K-10^f^, and other)	N/A^g^
Dagöö et al, 2014 [23]	Social anxiety disorder (R)	MINI phone interviews SCID^h^	*Open (2011 and 2012);* advertising in media and Facebook.	*Questionnaire* (LSAS-SR^i^)^a^	Internet platform
Gustafson et al, 2014 [24]	Alcohol use disorder (C^j^)	From treatment centers (DSM-IV^k^)	*Closed (17 months)*; from 3 nonprofit residential treatment centers	Risky drinking days^a^	Phone interview
Ly et al, 2014 [25]	Major depressive disorder (R)	MINI phone interview	*Open (N/A);* advertising in national media	*Questionnaires* (PHQ-9 and BDI-II)^a^	Internet platform
Depp et al, 2015 [26]	Bipolar disorder (R+C)	Medical records + MINI interview	*Open (N/A);* online, self-help groups, outpatient clinics, and communities	*Clinical ratings* (MADRS^l^ and YMRS^m^)^a^	N/A
Faurholt-Jepsen et al, 2015 [27]	Bipolar disorder (R+C)	From outpatient clinic + SCAN^n^	*Closed (18 months);* recruitment from specialized hospital function	*Clinical rating* (Hamilton Rating Scale for Depression 17 item and YMRS)^a^	Paper
Ly et al, 2015 [28]	Major depressive disorder (R)	MINI phone interview + PHQ-9	*Open (N/A);* advertising in media (for patients’ self-identifying as depressed)	*Questionnaires* (BDI-II)^a^	Internet platform
Moëll et al, 2015 [29]	Attention-deficit/hyperactivity disorder. (R+C)	Medical records + DSM-IV phone	*Open (N/A);* by patient websites and Facebook	*Questionnaire* Adult Self-Reported Scale subscale^a^	Internet platform
Ivanova et al, 2016 [30]	Social anxiety/panic disorder (R+C)	MINI phone + questionnaires	*Open (2 months);* advertising in national medias	*Questionnaire* (Generalized Anxiety Disorder 7-item, LSAS-SR, + more a)^a^	Internet platform
Hildebrandt et al, 2017 [31]	Binge eating and bulimia (R)	SCID interview + questionnaires	*Both (N/A);* community advertising and referrals	*Clinical ratings* (objective bulimic episodes Eating Disorder Examination)^a^	Paper and in-app
Mantani et al, 2017 [32]	Major depressive disorder (R+C)	Personal by treating physician	*Closed (25 months);* recruited by treating physicians	*Telephone* (blinded; PHQ-9)^a^	Telephone assessment
Ben-Zeev et al, 2018 [33]	Severe mental illness^o^ (C)	Chart diagnosis	*Closed (27 months);* identified from medical records, recruited by clinical teams	*Questionnaire* (Symptom Checklist-9) Engagement (*objective*)^a^	N/A
Boettcher et al, 2018 [34]	Social anxiety disorder (R)	DSM-IV (phone)	*Open (N/A);* advertising in national media, Facebook	*Questionnaire* (LSAS-SR)^a^	Internet platform
Bucci et al, 2018 [35]	Early psychosis (C)	From outpatient clinic	*Closed (7 months);* From early intervention for psychosis service	*Objective* measurements of feasibility and attrition^a^	N/A
Hur et al, 2018 [36]	Depression (other)^p^ (R)	SCID non-patient interview + questionnaires	*Open (N/A)*; advertising, online recruitment, posters and clinic	*Questionnaires* (Dysfunctional Attitude Scale)^a^	N/A
Liang et al, 2018 [37]	Substance use disorder (C)	From methadone treatment clinics	*Closed (11 months);* from methadone maintenance clinics and via social workers	*Robust objective measure* (drug use measured in urine)	Interviews
Schlosser et al, 2018 [38]	Schizophrenia spectrum disorders (R)	DSM-IV video-interview	*Open (N/A);* Craigslist, advertising, and information boards	Motivated behavior measured by trust task (objective task)^a^	Internet platform
Stolz et al, 2018 [39]	Social anxiety disorder (R)	DSM-IV (master students)	*Open (N/A);* media and online forums	*Questionnaires* (Social Phobia Scale, SIAS^q^, LSAS-SR)^a^	Internet platform
Faurholt-Jepsen et al, 2019 [40]	Bipolar disorder (R+C)	SCAN	*Closed (N/A);* patients previously treated in specialized function invited	*Clinical ratings* (HDRS-17 + YMRS)^a^	Paper
Krzystanek et al, 2019 [41]	Paranoid schizophrenia (C)	N/A (enrolled from treatment centers)	*Closed (7 month);* enrolled from 27 treatment centers	Many outcomes; clinical ratings (video; eg, Positive and Negative Syndrome Scale)	N/A
Stiles-Shields et al, 2019 [42]	Depression (R)	Quick Inventory of Depressive Symptomatology + MINI phone interview	*Open (5 month);* Craigslist	*Questionnaires* (eg, PHQ and usability)	Internet platform
Teng et al, 2019 [43]	Generalized Anxiety Disorder (R)	Questionnaire + DSM-IV subscale	N/A	*Questionnaires* (State-Trait Anxiety Inventory, BDI-II + more)	N/A
*Enock et al, 2014* [44]	Social anxiety disorder (R)	Questionnaire with cutoff	*Open (40 months);* news articles, message boards, craigslist.org, Google	*Questionnaire* (LSAS-SR, SIAS-17, and other)	Internet platform
*Roepke et al, 2015* [45]	Depression (R)	Questionnaire CES-D^r^ above 16	*Open (5 months);* Website and Craigslist	*Questionnaire* (CES-D at posttest)^a^	Internet platform
*Miner et al, 2016* [46]	PTSD^s^ (R)	Questionnaire PCL-C^t^ >30	*Open (16 months);* flyers, websites, Craigslist	*Questionnaire* (PCL-C) + feasibility and acceptability	Internet platform
*Possemato et al, 2016* [47]	PTSD (R+C)	Screened for PTSD + PCL >40	*Closed (4 months);* from veteran care unit, screened for PTSD.	Feasibility metrics + *Questionnaires* (PCL-S^u^, PHQ-9)	N/A
*Kuhn et al, 2017* [48]	PTSD (R)	Questionnaires PCL-C>34	*Open (10 months);* flyers, media, social media, Craigslist	*Questionnaire* (PCL–C)^a^	Internet platform

^a^Well-defined hierarchy in outcome measures.

^b^R: research based.

^c^MINI: Mini International Neuropsychiatric Interview.

^d^PHQ-9: Patient Health Questionnaire-9.

^e^BDI-II: Beck Depression Inventory.

^f^K-10: Kessler Psychological Distress Scale.

^g^N/A: not applicable.

^h^SCID: Structured Clinical Interview.

^i^LSAS-SR: Liebowitz Social Anxiety Scale—self-reported.

^j^C: clinical based.

^k^DSM: Diagnostic and Statistical Manual of Mental Disorders.

^l^MADRS: Montgomery–Åsberg Depression Rating Scale.

^m^YMRS: Young Mania Rating Scale.

^n^SCAN: Schedules for Clinical Assessment in Neuropsychiatry.

^o^Schizophrenia, schizoaffective disorder, bipolar disorder, and major depressive disorder.

^p^Other specified depressive disorder.

^q^SIAS: Social Interaction Anxiety Scale.

^r^CES-D: Center for Epidemiologic Studies Depression Scale.

^s^PTSD: posttraumatic stress disorder.

^t^PCL-C: Post-Traumatic Checklist—Civilian.

^u^PCL-S: Post-Traumatic Checklist Scale.

### Interventions

Although the included trials shared the concept of smartphones as a core feature of their interventions, the interventions were very different and tested in diverse settings with heterogeneous patient groups. Generally, 8 trials tested the app as a stand-alone treatment [35-37,44-48]. The remaining trials used variations of blended treatment and clinical support. Overall, 18 trials used prompts to engage users either from the app or by the investigators [22,26-28,30,32-44].

A glimpse of the diversity of interventions is presented here. Some trials used smartphones as a part of internet-based therapy, whereas others used smartphones as augmentation for face-to-face therapy or standard treatment programs. Most trials developed specific apps, whereas others used either commercially available or previously developed apps. Some interventions were interactive, whereas others had more static content. Some of the interventions made use of the unique possibilities a smartphone represents such as using global positioning system or allowing to interact with peers, relatives, or professionals, whereas others resemble classic internet therapies made for small smartphone screens to be more convenient and accessible to patients.

### Control Group

One of the included trials involved the use of a placebo app, consisting of an inactive version of the software with limitations, but with no further description [41]. In addition, 2 trials (that both tested the effect of attention bias modification via a dot probe on the smartphone screen) used a different version of the app for the control group, setting the dot appearance at random instead of following a predefined pattern that was central to the treatment [43,44]. In 1 trial on bipolar disorder, the participants in the control group received a *placebo* smartphone without the app system [27]. No other trials mentioned attempts on placebo treatment. Overall, 5 trials used standard treatment as the comparator [24,27,35,38,40], and 11 trials used a waitlist control group [29,30,34,38,39,41,42,44-46,48]. Furthermore, 4 trials used some sorts of clinical intervention as the comparator [26,28,31,33], and 5 trials compared the intervention with another app [23,25,35,36,42].

Further description of the smartphone-based treatment interventions, availability of technology, and author affiliation with industry can be found in Table 3 and is summarized in Table 4.

**Table 3:** List of randomized controlled trials involving smartphones in the field of psychiatry identified in systematic search updated in August 2019. Description of intervention and control group as well as authors cooperation with the industry. The bottom 5 trials in *italics* indicate trials with diagnoses solely based on questionnaires.

Author, year of publication	Short description of the intervention and main components. If available, the app name is displayed in italics.	Comparator. treatment received by the control groups	Blended treatment (BT)/app alone (AA)	TAU^a^	Cooperation/affiliation with the industry^b^	Description of technology available for the reader
Watts et al, 2013 [22]	CBT^c^-based “get happy program” with comic book–like lessons + homework activities	PC version of the same program	BT (limited clinician contact)	N/A^d^	No	Brief description and few screenshots
Dagöö et al, 2014 [23]	Guided internet-based CBT adapted for mobile phone administration	Another app similar therapist contact	BT (limited clinician contact)	No	N/A	Brief descriptions
Gustafson et al, 2014 [24]	*A-chess:* app with static self-help content and interactive features with therapist feedback	TAU	BT	Yes	No	App fully available online and a description of the app is attached
Ly et al, 2014 [25]	App delivering behavioral activation psychotherapy with possible but limited clinician contact	Mindfulness app, similar therapist contact	BT (limited clinician contact)	No	The first author has a similar app on the open market	Good descriptions and screenshots
Depp et al, 2015 [26]	*PRISM:* interactive monitoring and intervention linking mood and activities with self-management strategies	Active control monitoring on paper	BT	N/A	No	Thorough descriptions but no technical reports or screenshots
Faurholt-Jepsen et al, 2015 [27]	*MONARCA:* self-monitoring with a double feedback loop between clinic (nurse) and patient	TAU + nurse contact + phone without app	BT	Yes	No	Thorough descriptions and screenshots in the protocol
Ly et al, 2015 [28]	Four therapy sessions and a smartphone app, based on behavioral activation, used between sessions	Full behavioral activation (10 sessions)	BT	No	N/A	Brief descriptions and screenshots
Moëll et al, 2015 [29]	*Living smart:* Guided online course to structure life using multiple already available apps	Waitlist control	BT	No	N/A	Multiple already-available apps
Ivanova et al, 2016 [30]	Internet therapy + an app^e^ promoting change corresponding to the core treatment program, with therapist support	Waitlist or intervention without therapist support	BT	No	2 authors employed by a technology company; 1 developed a similar app	Description and screenshots available in the protocol
Hildebrandt et al, 2017 [31]	*NOON self-monitoring:* App as an augmentation of traditional guided self-help	Guided self-help therapy without an app	BT	No	3/5 authors have a connection to NOOM who developed the app	Short descriptions and no screenshots
Mantani et al, 2017 [32]	*Kokoro:* CBT-based self-help app with 8 sessions presented by cartoons + fixed-dose medicine shift	Medicine shift with fixed dose and no app	BT^f^	No	2 of the authors developed the app	A thorough report describing the app in detail
Ben-Zeev et al, 2018 [33]	*FOCUS:* Multimodal smartphone intervention including self-assessments and on-demand functions	Clinic-based group intervention	BT	No	First author had a consulting agreement with technology company	A short description in the text - further in supplement
Boettcher et al, 2018 [34]	*Challenger:* App promoting exposure exercise through interactive challenges + internet-based therapy	2) Waitlist control or 3) internet therapy alone	BT	No	The third author founded the app company	A short description + referral to further information
Bucci et al, 2018 [35]	*Actissist:* Self-help app that asks questions and has automated responses and various static supportive content	TAU + another app	AA	Yes	N/A	Descriptions of app and screenshots
Hur et al, 2018 [36]	*TODAC:* A scenario-based CBT app to reduce dysfunctional beliefs	Mood diary app	AA	N/A	No	Descriptions of the app modules, and small screenshots
Liang et al, 2018 [37]	*S-health:* Simple smartphone app that sends messages, controls cravings, and has a survey	Receiving text messages about various topics	AA	Yes	N/A	Survey and screenshots available + short description
Schlosser et al, 2018 [38]	*Prime:* Personalized real-time intervention for motivational enhancement. App-based online community	TAU/waitlist control	BT	Yes	No	Short description, but no screenshots
Stolz et al, 2018 [39]	Mobile version of validated psychoeducative self-help program with 8 modules based on cognitive therapy	Waitlist control or PC version	BT (limited clinician contact)	No	N/A	Short description of modules, but no screenshots
Faurholt-Jepsen et al, 2019 [40]	*MONSENSO:* Self-monitoring + objective monitoring with a double feedback loop between nurse and patient	TAU + offer to borrow a smartphone	BT	Yes	2 coauthors are shareholders in Monsenso	Thorough descriptions and screenshots in the protocol
Krzystanek et al, 2019 [41]	*MONEO:* Medication reminder, cognitive training, information bank, and “tele visits” with the investigator	Inactive version + monthly video examination	BT	N/A	No	Refers to online supplementary that was not possible to find
Stiles-Shields et al, 2019 [42]	*Boost Me* (behavioral app) or *Thought Challenger* (a cognitive app): Both with brief weekly coaching	2 different apps and 1 waitlist control	BT (with limited coaching)	No	Last author has an ownership interest in Actualize Therapy	Both apps are available free online
Teng et al, 2019 [43]	Home-delivered attention bias modification training with dot probe on screen	Control group with random dot or waitlist	BT	N/A	No	Short descriptions and few screenshots
*Enock et al, 2014* [44]	Cognitive training via smartphone with attention bias modification training	An active control group and waitlist control	AA	Yes	No	Thorough descriptions, links, and few screenshots
*Roepke et al, 2015* [45]	*SuperBetter:* Self-help game using either specific CBT or a general version using self-esteem and acceptance	2 versions of the app and 1 waitlist control	AA	Yes	3 authors work for SuperBetter (1 founded it)	Short description and 2 screenshots
*Miner et al, 2016* [46]	*PTSD coach:* Psychoeducation, symptom assessments, self- management + access to supportive others	Waitlist control	AA	N/A	N/A	The app is available free online
*Possemato et al, 2016* [47]	*PTSD coach:* Multifunctional psychoeducative self-help app with clinical support of 4, 20-min sessions	App alone	BT<=>AA	No	No	The app is available free online
*Kuhn et al, 2017* [48]	*PTSD coach:* psychoeducation, symptom assessments, self-management + access to supportive others	Waitlist control	AA	No	N/A	The app is available free online

^a^TAU: Treatment as usual.

^b^Information assessed by author affiliation, grand support, and conflict of interest.

^c^CBT: cognitive behavioral therapy.

^d^N/A: not applicable.

^e^Two treatments not technologically attached but based on same therapy.

^f^Allowed to discuss the app with treating physician.

**Table 4:** Summarized findings from systematic review on 27 randomized controlled trials involving smartphones in the field of psychiatry. Consolidated Standards of Reporting Trials electronic health checklist is used as a guideline to systematically display trial design, methodology, and reporting of the identified trials.

*Consort item*	*Summarized findings according to CONSORT item, with references to relevant articles.*
Title and abstract (1a and 1b)	All but 6 titles [22,35,37,38,41,42] described the mode of delivery, components of treatment, target group, and trial design according to Consolidated Standards of Reporting Trials electronic health guidelines [20]. Often only broad terms of components were used, such as “mobile” or “mHealth.”
Introduction (2a and 2b)	The trials were published from 2013 to 2019 with equal distribution through 2013 to 2017 and increasing numbers from the from 2018 and forward [33-43]. Trials were mainly from western countries, especially from Scandinavia [23,25,27-30,34,40].
Trial design (3)	A total of 19 trials were classic RCTs [23,25-27,29-34,38-41,43-45,48], 7 were pilot RCTs [22,35-37,42,46,47], and 1 was a noninferiority RCT [28].
Participants (4a and 4b)	A total of 22 trials used research-based diagnoses: 5 were based on questionnaires [44-48], 8 used phone interviews [22 25,28-30,34,42], 1 used Facetime or Skype interview [38], and 8 used personal interviews [26,27,31,32,36,39,43] mostly based on either MINI or DSM-4; 5 trials based their diagnoses only on clinical-based information [24,33,35,37,41]; 13 trials excluded patients with various degree of suicidal ideation [22,23,25,28-32,34,36,39,42,47]; 3 trials excluded patients with too severe symptomatology within the diagnosis of interest [22,26,27]. Most trials excluded patients with severe psychiatric comorbidity from lower International Statistical Classification of Diseases and Related Health Problems-10 chapter. In addition, 12 trials supplied participants with smartphones, either voluntary or mandatory [24,26,27,31,33,35,38,40,41,46-48]; 12 trials compensated participation in assessments with money or gift cards [26,33-38,42,43,46-48].
Interventions (5)	Intervention length varied substantially: from 3 weeks [36] to 52 weeks [41]. Most interventions lasted between 4 and 12 weeks; 8 trials used unaffected standard treatment beside intervention [24,27,35,37,38,40,44,45]; 8 trials tested the app alone [35-37,44-48], the remaining used variations of blended treatment; 1 trial tested blended therapy against app alone [47]; 18 trials used prompts to engage users, either from the app or by investigators [22,26-28,30,32-44]; 1 trial compared with an inactive “placebo” version of the app [41]; 2 trials compared with a placebo training module [43,44]. In 1 trial, participants in the control group received a “placebo” smartphone without the app system [27]; 5 trials used standard treatment as comparator [24,27,35,38,40], 11 trials used waitlist control [29,30,34,38,39,42-46,48], 4 trials used clinical intervention [26,28,31,33], and 5 trials compared with another app [23,25,35,36,42]; 1 trial collected automatically generated data [40] and further 8 trials collected data on app usage [24,33,34,42,43,45,47,48]; in 3 trials, intervention was only for iPhone [32,34,45] and in 3 only for Android [27,42,43]. The rest of the trials either had a Web-based version available or app for both platforms. Only 1 article mentioned information about updates of apps or intervention [27].
Outcomes (6a and 6b)	Overall, 8 trials did not use a predefined hierarchy of outcome measures [22,37,41-44,46,47]; 1 trial used tested levels of drug use in urine as a specific detection [37]; 1 trial used objective measurements of feasibility, use, and attrition as the primary outcome [35]; 1 trial tested a specific task [38]; 1 trial used video call–based clinical ratings [41]. Only 4 trials used clinical ratings as the primary outcome [26,27,31,40]. Remaining trials used patient-reported outcome measures. A total of 12 trials used internet platform for data collection [23,25,28-30,34,38,39,42-46,48], with 6 of these mentioning validations of questionnaires for online use [23,25,28,30,34,46]; 2 trials used the app for outcome measure [31,41].
Sample size (7a and 7b)	Sample size varied from 20 participants [47] to 429 participants [44]; 11 trials with numbers above 100 participants [24,30,32-34,39-41,44,45,48]. Pilot trials were smaller.
Randomization (8, 9, and 10)	Overall, 8 trials did not supply information about randomization [29,31,36-38,45-47]; 1 used Excel [43], and 1 used the app for randomization [41]. The remaining mainly used online software.
Blinding (11a and 11b)	Overall, 2 trials claim to be double-blinded with no further explanation on how blinding was assured [43,44]; 1 trial blinded app allocation for the patients (they tested 2 different apps) [45]. The remaining trials had no blinding of patients. In 12 trials, authors explicitly wrote that they used blinded assessments for outcome measures [25-29,31-33,35,38,40,44]. Within these 12 trials, 5 trials used patient-reported outcome measures as the primary outcome measure with nonblinded patients [25,28,29,32,33]; 1 trial tested for the success of blinding [32].
Statistical methods (12a and 12b)	A total of 11 trials based sample size on power calculations [24,27,28,30-33,39,40,45,48]. All but 1 of these managed to recruit at least the desired number [31]. No trials took changes and updates of software or technical problems into account in statistical methods.
Participant flow (13a and 13b)	All but 2 trials [43,44] presented a trial flow chart of eligible subjects, although with various details on reasons not to participate and drop out. Completion rates were reported very differently, varying from 163/164 completing primary outcome [32] to 74/283 completing posttreatment assessments (primary outcome) [45]. All but 2 trials reported on adherence to treatment [36,43].
Recruitment (14a and 14b)	Recruitment length was reported in 16 trials and varied from a few months to several years [22-24,27,30,32,33,35,37,41,42,44-48]; 10 trials used closed recruitment with a referral from clinicians or researchers seeking out participants from a well-defined patient population [24,27,31-33,35,37,40,41,47]; 1 trial gave no information on recruitment [43]. The remaining trials used open recruitment mainly via Craigslist.org, advertising in traditional ways, or through social media.
Baseline data (15)	Only 2 trials included technology-specific baseline data or information about participant technological abilities [32,33].
Numbers analyzed (16)	All but 6 trials [22,36,37,41-43] used the intent-to-treat principles in the primary analysis.
Outcome and estimation (17)	A total of 17 trials presented intensity of use or user data, either in the article or in supplementary data, with significant variations in usage among subjects and between trials [22-25,27,32-35,37-40,42,43,45,47].
Harms (19)	Overall, 5 trials prospectively measured harms or adverse events and reported directly in paper [32,33,35,41,42]. These trials found no harm from smartphone treatment used. One of these trials [42] had a safety protocol with clear, standardized instructions on how to react to suicidal ideation; 1 trial found a negative effect of treatment in secondary analysis, indicating fewer improvements in symptoms in a subgroup with a higher baseline score on the Hamilton Rating Scale compared with controls [27].No trials mentioned privacy breaches. Three trials mentioned technical problems and how these affected the intervention [23,40,48].
Generalizability (21)	Trials were heterogeneous. Some had strict criteria on diagnosis, comorbidity, and ongoing treatment, whereas others were pragmatic trials with few exclusion criteria. Trial populations varied from patients recruited among the general population who might not have sought help in the regular treatment system [22,23,25,28,30,34,39,42,44], whereas others came from specialized clinical functions setups [24,27,31-33,35,37,40,41,47].
Registration (23)	A total of 14 articles included information about trial registration [22,24-27,29-33,35,39-41].
Protocol (24)	A total of 5 trials published their trial protocol [27,30,32,35,40]; 1 trial had the protocol attached to the publication [28].
Funding (25)	Most authors came from universities; 15 trials reported information regarding funding [24-28,31,32,35-38,40,41,43,47] with funding mainly coming from public funds and institutions.
Competing interest (X27)^a^	A total of 9 articles declared having various degree of affiliation with private technology companies or closed relation to the app that they tested [25,30-34,40,42,45]; 8 trials did not include conflicts of interest in the printed article [23,28,29,35,37,39,46,48].

^a^Not an original Consolidated Standards of Reporting Trials item but included in the Consort electronic health checklist as *X27*.

### Adherence to Smartphone Intervention

All but 2 trials [36,41] reported adherence by using one of the following or a combination: report on how many in-app lessons, modules or sessions participants completed, the number of daily or weekly users or logins, numbers of active users at a given time point, and composite scores measuring adherence.

Some trials included large tables on the usage of different components of the app, for example, the study by Schlosser et al [38], or included detailed program usage in the supplementary section, for example, the study by Stolz et al [39].

One trial economically compensated the participants for using the app [35]. Further comparison of adherence between trials was not feasible because of significant differences in how adherence was collected and reported in the individual trials.

### Statistical Power Analysis

Overall, 11 trials based their required sample size on power analyses [24,27,28,30-33,39,40,45,48]. All but 1 [31] of these trials managed to recruit at least the desired number. In addition, 6 of the trials without power analyses were specified as pilot studies [22,35-37,42,46,47].

### Statistical Analysis

All but 6 trials [22,36,37,41-43] used intent-to-treat principles in the primary analyses. Different methods were used to account for missing data. Some trials used multiple imputations [23,46,48], whereas others used mixed models [22,24,27,28,30,32,34,39,40,45] with *missing at random* or maximum likelihood estimations. One trial omitted data without imputation [41]. In addition, 8 trials included no explicit information on how the authors handled missing data [33,35-38,42,44,47]. Furthermore, 4 trials referred to an available predefined plan of analyses in the statistical section [27,32,33,35].

### Technical Aspects of Smartphone-Based Treatment Interventions

One article informed about technical updates of the app, changes in interventions because of improvements, or technological problems that might have influenced the intervention over time [27]. None of the trials mentioned privacy breaches. In addition, 3 trials mentioned technical problems and how these affected interventions and the main hypothesis [23,40,48]. One trial collected automatically generated smartphone data (phone usage, social activity, and mobility) [40]. Furthermore, 8 trials collected data on app usage [24,33,34,42,43,45,47,48]. None of the trials accounted for changes or updates in the software or technical problems related to the intervention in the statistical analyses. Two trials included technology-specific baseline data or information about the participant’s technological abilities such as prior smartphone use or assessments of the participant’s ability to use a smartphone [32,33]. A total of 17 trials presented the intensity of use or user data with significant variations in usage among subjects and between trials [22-25,27,32-35,37-40,42,43,45,47].

### Ethical Aspects

None of the included trials addressed the potential ethical aspects of using smartphones in the treatment of patients with a psychiatric disorder. Two trials included a section on ethics, including information on various trial registrations and approvals, data storage, and economic compensation [27,40].

## Discussion

### Principal Findings

This is the first systematic review regarding methodological challenges in RCTs investigating smartphone-based treatment interventions in patients with a psychiatric diagnosis. We included 27 trials with a wide range of psychiatric diagnoses and observed substantial between-trial heterogeneity. The trials were conducted in diverse settings and used different smartphone-based treatment interventions and different follow-up periods. The trials reported on various outcome measures, which, in nearly half of the trials, were not clearly predefined. Most trials only used unblinded patient-evaluated outcome measures. A single trial reported on the success of blinding procedures.

Furthermore, only 1 trial provided information regarding technological updates of the smartphone-based treatment intervention. A declaration of interests was missing in 9 of the trials. No trial compared participants with nonparticipants, thereby increasing the risk of selection bias and making generalization of the trial findings difficult.

The included trials used smartphones in various ways and applied treatments to very heterogenic populations. Generally, the combination of insecure diagnoses, lack of blinding, use of patient-evaluated outcome measures, and lack of trial protocols or thorough publicly available trial registrations implies that evidence on the effects and side effects is still warranted.

The included trials used very different comparators, and the treatments given, besides the intervention of interest, varied from nothing to intensive clinical standard treatment setups. Generally, important aspects concerning technological features and how these inevitably affect outcomes were sparingly reported. On the basis of the results of the review, in the following sections, we discuss the highlights and suggest recommendations for designing and conducting future RCTs investigating the effect of smartphones in psychiatry.

### Inclusion Criteria of Trial Patients

Owing to the lack of diagnostic biomarkers within psychiatry, currently, the research-based clinical diagnostic process represents the golden standard. The use of online, patient-evaluated diagnoses will reduce the validity of the diagnoses and reduce the generalizability to the clinical practice of trial results. 

The use of a research-based clinical diagnostic assessment such as the Schedules for Clinical Assessment in Neuropsychiatry providing Diagnostic and Statistical Manual of Mental Disorders and ICD-10 diagnosis [49] or another systematic diagnostic assessment system should be prioritized. If patients are thoroughly and validly diagnosed and characterized before inclusion in the RCT, that is, by their treating doctors or psychologists, the clinical diagnoses may as well be used, depending on the aims and hypotheses of the RCT.

Smartphones allow for a vast amount of data to be collected online or automatically. This could be used as an advantage when conducting large RCTs. Digital solutions let researchers reach more participants and make it easier for patients to participate in clinical trials, not having to show up in the research settings for diagnostic assessment, baseline data collection, and outcome assessments. This possibly facilitates broader and more feasible inclusion of patients. Questionnaires conducted on the screen of a smartphone might vary compared with validated paper-based questionnaires. However, a Cochrane review from 2015 concludes that “apps might not affect data equivalence as long as the intended clinical application of the survey questionnaire, its intended frequency of administration and the setting in that it was validated remain unchanged” [50]. Telephonic interviews are different from clinical evaluations conducted by clinicians with possibly more reduced validity of diagnoses and sociodemographic and clinical data. Nevertheless, telephonic interviews (by trained lay interviewers or professionals) have been used in the research of depression and anxiety disorders with reasonable validity of diagnosis [51].

Modern electronic data collection should be used wisely with an awareness of possible changes in the quality of the data that the researchers obtain.

### Interventions and Comparators

This systematic review showed that a placebo-controlled design was used to a limited extent. There is no clear definition of what a digital placebo treatment can or should contain, and defining a proper placebo group in a nonpharmacological RCTs is always difficult. The expectation for technical solutions themselves to be helpful resembles a reaction that is comparable to receiving a placebo pill and has been suggested to affect patients independent of active treatment [52]. The term *digital placebo* has been suggested [52]. Still, little is known about this issue; however, ongoing RCTs are investigating this subject further using a sham app as a comparator [53].

Clear descriptions of the content of the interventions and comparators used by researchers in future RCTs should be prioritized and made available to readers. If not mentioned in the primary publication, clear reference to the description should be made, such as in the study protocol, earlier publication, appendices, or publicly available versions of the intervention used. Furthermore, researchers could beneficially design the active intervention to fit into clinical practice and adapt to the clinical settings either as a stand-alone treatment or in combination with clinical treatment and support.

### Outcome Measures and Power Analyses

A clear predefined research question, represented by a predefined primary outcome measure in a precisely well-defined patient population, is necessary. Selecting a predefined and relevant primary outcome measure is crucial [12]. Assessors should be blinded to outcome measures. End points of clinical relevance, that is independent of or blinded to researchers, such as admittance to a hospital or relapse or recurrence of illness (as in an ongoing trial [54]), should be prioritized compared with biased end points [12]. Such outcomes benefit from being critical for patients, relatives, and clinicians.

Power analyses should preferably be made before start the trial to ensure that the required sample is realistic and able to answer the primary research question—often represented by significant changes in the primary outcome.

When using clinician-based outcome measures, this should preferably be done by blinded trained clinical researchers. This is a difficult task because patients may give the researcher a hint on their allocation. Precautions to hinder this should be taken and described.

Although patient-evaluated outcome measures are appealing because of the ease use, we should be careful when interpreting findings based on unblinded patient-evaluated outcomes as there will be a risk of bias.

Clinicians and administrators may play important roles in implementing such treatment systems following trial findings if the results indicate a possible effect of the new treatment [55].

Possible outcomes could include measures of clinicians’ and caregivers’ attitudes toward the intervention if involved. Further data regarding the use of resources and economic costs related to the intervention could be included if available.

### Publication of a Trial Protocol and Reporting Guidelines

Compilation and publication of a trial protocol or a thorough description on publicly available registration sites, such as https://clinicaltrials.gov/, before analyzing data from the trial will increase the validity of the findings. Trial protocols and registrations should follow the standards for medical trial protocols [20] such as precise descriptions of inclusion and exclusion criteria, recruitment procedures, prioritized outcome measures including unblinded assessment, statistical power analyses, and a plan for analyses of participants versus nonparticipants. Furthermore, a thorough description of the used technology should be included [54,56,57]. Authors should publish deviations on especially the technical side of the protocol that were necessary, including valid arguments for the changes.

When reporting results, guidelines such as the CONSORT eHealth checklist [20] should be followed to increase transparency and for evaluation of findings.

### Multidisciplinary Research

A close collaboration between information technology developers, clinicians and researchers is critical to ensure that the technology developed can answer the primary questions that are addressed and, vice versa, that the posed hypotheses match the available technology.

Authors should use the same transparency when working with the technology industry as researchers, doctors, and funders use with the medical industry. This systematic review shows that the authors’ declarations of conflicts of interest are frequently missing. The industry has a significant direct or indirect interest in developing app solutions and gathers information from patients as information in the digital economy involves major economic interests [58]. Any potential conflicts of interest should always be declared.

### Technology in Randomized Controlled Trials

Smartphones represent a unique tool to measure adherence and fidelity in RCTs. However, there is no clear definition of how to measure or report adherence to smartphone-based treatment systems.

Smartphone solutions might assist in follow-up assessments and could advantageously use built-in features to ease the patients. Even when patients are “lost to follow-up,” assessments based on smartphone sensors could be collected, with patient permission, and might reflect behavioral changes for the user [59-61].

Prompts and reminders may comprise an important part of digital solutions to keep patients engaged in treatment.

These prompts, preferably, should be a part of the intended intervention that is investigated and not done only as part of the research project. The research team will potentially not be part of a real-world implementation of the technology and therefore give a false picture of adherence and effect of the intervention.

Information about technical skills and smartphone usage and habits might be valuable information and should be presented.

Reporting possible harms and adverse events are essential as the field of smartphone-based treatment is new, and little is known of potentially harmful effects such as worsening of symptoms or suicidal ideation. One trial has reported possible harms of using the intervention [27].

Potential harms and safety matters should be taken into consideration when designing and conducting the trial, as the example of the study by Stiles-Shields et al, [42] in which a safety protocol was used to standardize how researchers should react on suicidal ideations, expressed by participants either in the app or as part of the online questionnaires (where suicidal thoughts is a common item).

### Updates, Revisions, and Adaptive Trials

Developing well-designed RCTs, including proper diagnostic procedures and thorough, robust, and blinded outcome measures, will inevitably be a time-consuming process. Still, it is crucial to test new smartphone-based treatment interventions on well-defined patient populations. *Locking* a digital intervention for several years is a challenge because technology would be expected to evolve and improve over time. In software engineering, updates and enhancements of the software are frequently released to fix bugs and errors and to improve usability, stability, robustness, and security. Moreover, the type of data collected from the phone via sensors and its processing by algorithms will also constantly change and adapt according to automatic machine learning models. Such learning systems will change based on new information and might be used increasingly in psychiatry as well as in many other fields [62]. Finally, the hardware and operating systems of the mobile phone regularly change, and the major vendors (Apple and Google) regularly release new hardware and operating systems that affect what an app can and cannot do.

Consequently, the concept of maintaining the digital intervention stable during a clinical trial is unrealistic, taking into consideration the long time span involved in conducting most RCTs.

To merge these opposing interests, we must accept these ongoing changes as a natural consequence of conducting research in the field, similar to surgeons who improve their skills or therapist who adapt and improve as they see more and more patients. Such updates should be reported systematically and should be thoroughly described so that it will be clear to readers which type of updates were made and what effect they might have had on trial results. In addition, the possible effects of add-ons could be presented and provided enough statistical power for subanalyses.

By allowing a more flexible approach to the RCT design, it would conform more to the so-called adaptive trial paradigm [17,63], and then RCTs might be able to answer questions regarding the effect of mHealth interventions without evaluating outdated technology [16,17].

It is necessary to accept and incorporate technological developments and changes in the design of a specific RCT in line with suggestions from similar but older scientific fields as telehealth in general [64].

### Ethical Aspects

In a digital economy, data generated by apps and services constitute a valuable resource. Within psychiatry and other areas, such data can lead to discrimination of individuals and patient groups as a whole, and therefore, the handling and use of the data collected are of great importance. The value such information has to industry and other stakeholders is driving the many apps that are widely available without evidence of effects or possible harms. We need information obtained through well-designed and transparent trials to improve our knowledge of how these treatments can help patients and professionals.

Privacy, security, technology illiteracy, depersonalization of treatment systems, and technological paternalism are some of many possible ethical issues in this field [58].

These challenges apply both to the trial designs and the treatments being developed. The included trials hardly mentioned any such ethical issues.

Trials must consider and discuss possible ethical implications of the trial design and of the treatment itself.

### Findings in a Scientific Context

The results from one of the included trials have been replicated using the same intervention, trial design, and outcome measures in a second trial only once [27,40]. However, this is necessary to increase evidence. Notably, numerous trial protocols within the field were identified during the search [54,65-69]. This indicates that the pace of the field is still increasing, and hopefully, future trials will provide more evidence within the field of smartphone-based treatment in patients with psychiatric disorders. This development is further shown in the large numbers of RCTs found in this review. Only 1 of the included trials in this review was published and included in a previous review from 2013 investigating the effects of smartphones in mental health [18]. With an increasing number of RCTs and hopefully increasing the quality of design and method, and a more uniform reporting of results, future meta-analyses of the effects of treatments will be possible.

### Limitations

This study has various limitations: not all parts of the review process and data collection were double checked, no protocol of study method was published online beforehand, and no meta-analyses or statistical analyses were performed on the included data. We did not use any scoring systems for the assessments of quality and risk of bias in the included studies, leaving the assessment to the reader.

### Conclusions

This first systematic review on the design, conduct, and methodological challenges of RCTs investigating the effect of smartphone-based treatment in patients with a psychiatric diagnosis suggests that there is a rapidly increasing interest for this type of treatment. Although an increasing number of trials tested new smartphone-based treatments, the trial designs and reporting were of low quality compared with more classic medical RCTs, and heterogeneity and methodological issues in individual trials limit the evidence.

Smartphone-based treatment interventions imply new challenges and opportunities, but we, as researchers, should consider strict methodological efforts when designing, conducting, and reporting such trials as in the rest of the field of medicine. Future trials employing strict methodology, including detailed description regarding patient recruitment, pre- and well-defined, prioritized outcome measures, information regarding technical updates and down periods, and statements on potential conflicts of interest are warranted. Research groups without trial experience should seek out information on how to conduct RCTs with high a methodological standard to ensure a high level of quality in the research. Finally, close collaborations between professions and specialties are needed in this complex branch of science.

## Appendix

Multimedia Appendix 1 Data extraction template.

## Abbreviations

BDI-II: Beck Depression Inventory
CBT: cognitive behavioral therapy
CES-D: Center for Epidemiologic Studies Depression Scale
CONSORT: Consolidated Standards of Reporting Trials
DSM: Diagnostic and Statistical Manual of Mental Disorders
eHealth: electronic health
ICD-10: International Statistical Classification of Diseases and Related Health Problems-10
K-10: Kessler Psychological Distress Scale
LSAS-SR: Liebowitz Social Anxiety Scale—self-reported
MADRS: Montgomery–Åsberg Depression Rating Scale
mHealth: mobile health
MINI: Mini International Neuropsychiatric Interview
NI: noninferiority
PCL-C: Post-Traumatic Checklist—Civilian
PCL-S: Post-Traumatic Checklist Scale
PHQ-9: Patient Health Questionnaire-9
PTSD: posttraumatic stress disorder
RCT: randomized controlled trial
SCAN: Schedules for Clinical Assessment in Neuropsychiatry
SCID: Structured Clinical Interview.
SIAS: Social Interaction Anxiety Scale
TAU: Treatment as usual
YMRS: Young Mania Rating Scale
