# Supplementary material for: Methodological Challenges in Randomized Controlled Trials on Smartphone-Based Treatment in Psychiatry: Systematic Review
Source: J Med Internet Res. 2019 Oct 27;21(10):e15362. doi: 10.2196/15362 (PMC6914239; doi:10.2196/15362)
Supplement: Multimedia Appendix 1 [file jmir_v21i10e15362_app1.pdf]

Titel and abstract (1a and 1b)	• (Fulfilling consort recommendations?)
Introduction (2a and 2b)	• Year of publication<br>• Country<br>• Author<br>• journal
Trial design (3a and 3b)	• Trial design *(pilot study/feasibility study?) Pilot ⇔effect RCT*<br>• Allowing updates/changes during trial? (*As part of design)*<br>• *Pragmatic trial? (mentioned by author)*<br>• *Published design report? (different from protocol?*<br>• *Investigator initiated ⇔ industry initiated*
Participants (4a and ab)	• Baseline: age, nationality, gender, diagnose<br>• How is diagnose obtained*: interview? Questionnaire-based? Research based ⇔ clinic based*<br>• Do they receive a telephone?
Interventions (5)	• Intervention length<br>• Description of intervention<br>• Specific or broad intervention => are they measuring a APP or content of an app?<br>• Blended treatment / cointerventions or app alone?<br>• Use of standard treatment for everyone?<br>• Comparator*: TAU, other APP, Placebo APP, Other treatment?*<br>• Type of intervention *(game, monitoring? information,)*<br>• Use of prompts?<br>• Device used: *tablet, desktop in addition to smartphone*<br>• Platform used? (*apple, android etc.)*<br>• free use/time fixed?<br>• Clear mention of revisions and updates *(Bug fixes, Downtimes, content change)* (minor or major changes?)<br>• Replicability possible by publishing source code, screenshots desvription etc.
Outcomes (6a and 6b)	• Primary outcome measure:<br>*independent robust outcompetes OR clinical OR questionnaire*<br>• Secondary outcome *(including how it is measured)*<br>*independent robust outcome measures OR clinical OR questionnaire*<br>• Data collection: paper questionnaires/digital questionnaires /questionnaires in app/ automatic data collection<br>• Questionnaires validated for online/electronic use

	• Qualitative feedback received?<br>• Well-defined appriori hierarchy in outcome measures
Sample size (7a and 7b)	• Sample size,
Randomization 8a, 8b, 9 and 10	• Allocation concealed? (ye/no)<br>• Independent (yes no)<br>• Random? (yes no)
Blinding (11a and 11b)	• Blinding: *Patient/researcher?*<br>• Researcher blinded to end of statistical analysis
Statistical methods (12a og 12b)	• Taking changes in intervention (updates, crashes etc.) into account?<br>• Power analysis made?
Participant flow (13a and 13b)	• Number lost after randomization?<br>• Adherence to treatment<br>• Adherence to study assessments<br>• IS there a description/flowchart?
Recruitment (14a and 14b)	• Length of recruitment period.<br>•<br>• Recruitment type: *Open vs closed. Web-based or face-to-face. (online/hospital/referrals/?)*<br>•
Baseline data (15)	• Baseline data containing technology-relevant data? *(access to internet, smartphone, experience with technology etc.)*
Numbers analyzed (16)	• Intension to treat-analyses
Outcome and estimation (17a and 17b)	• Presentation of intensity of use/user data<br>• Effect of treatment? *As reported in abstract*
Ancillary Analyses (18)	•
Harms (19)	• Harms reported?<br>• Harms measured<br>• Harms detected<br>• Privacy breaches<br>• Technical problems
Limitations (20)	•
Generalizability (21)	• Narrow or wide inclusion/exclusion (comorbidity, age, other problems, exclusion due to severity of disease.)

Interpretation (22)	•
Registration (23)	•
Protocol (24)	• Study protocol published?
Funding (25)	• Public or private?<br>• Company cooperation between academia and industry mentioned
